# Infra-low frequency neurofeedback and insomnia as a model of CNS dysregulation

**DOI:** 10.3389/fnhum.2022.959491

**Published:** 2022-09-21

**Authors:** Paul Terrence Moore

**Affiliations:** 4BetterSleep Center, Dallas, TX, United States

**Keywords:** infra-low frequency, neurofeedback, insomnia alternative treatments, dysregulation, sleep disturbance, infra-low frequency neurofeedback

## Abstract

This paper will review what is conventionally known of sleep homeostasis and focus on insomnia as a primary manifestation of brain dysregulation, whether as a solitary symptom or as part of a larger syndrome such as post-traumatic stress disorder, PTSD. It will discuss in brief behavioral/mindfulness treatments that have been used to treat neurologic diseases, as this is germane to the phenomenology of neurofeedback (NF). It will explore how neurofeedback may work at the subconscious level and cover the current clinical experience of the effectiveness of this technique in the treatment of insomnia. It will conclude with a case presentation.

## Introduction

The brain may be considered a “self-regulating organism” inasmuch as it can operate autonomously. In other words, it can modulate its own activity (endogenous neuromodulation) in response to both internal and external stimuli. The “normal” brain can maintain stable functioning despite a wide range of operating circumstances or exigencies. A dysregulated brain manifests itself in manifold ways that may be categorized as disorders of arousal, instabilities, disinhibitions, localized dysfunction and learned, maladaptive behaviors. Allopathic medical therapeutics to date offers pharmaceuticals and/or invasive procedures which do not address underlying problems with dysregulation effectively, as indicated by the marginal improvements associated with these treatments. Exploiting neuroplasticity *via* endogenous neuromodulation may provide clinically relevant functional improvement to the many persons who suffer with a poorly regulated brain. Biofeedback in general, and neurofeedback in particular, have an established clinical record of affording such improvement.

## Dysfunctional sleep

Good sleep is central to normal wakeful functioning. Most have had practical experience of the effects of inadequate or disrupted sleep. Apart from the unpleasantness of the feeling of inappropriate sleepiness, it results in global impairment of cognitive functioning such as maintenance of attention, judgement, memory, motor control and emotional modulation. Modern society appears designed to disrupt the restorative properties of sleep through multiple mechanisms including sleep deprivation, sleep disruption and disturbance of circadian rhythms. For example, due to the electrification of human civilization, most people in developed countries are exposed to circadian altering light well past sundown. This same fact has also led to widespread sleep deprivation. Modern lifestyles promote maladaptive sleep habits and underpin many complaints of insomnia. Dysfunctional sleep has been linked to eating habits that promote weight gain. Furthermore, modern “food” directly promotes ill-health primarily through alteration of normal metabolism and promotion of unchecked weight gain, which itself results in chronic illnesses such as type II diabetes, cardiovascular disease, sleep-disordered breathing, and degenerative arthritis.

Clinical studies have demonstrated that sleep-deprived individuals have cognitive and motor impairment comparable to persons with blood alcohol levels of 0.1% (Williamson and Feyer, 2000). Chronic sleep impairment of any cause is associated with chronic health conditions such as hypertension, cardiovascular disease, and endocrine and immune dysfunction (Taylor et al., 2007). For example, sleep restriction in men results in increased numbers of circulating white blood cells which had not returned to normal after even 9 days of recovery sleep (Lasselin et al., 2015). Such effects on the immune system may be related to the development of such chronic disorders as type II diabetes (Tsujimura et al., 2009).

The importance of adequate sleep is underscored by the fact that longer sleep in obese persons with chronic sleep restriction predicts a reduction in BMI (Body Mass Index) (Verhoef et al., 2013). Sleep restriction has been associated with alterations in metabolism of lipids, neurotransmitters, stress mechanisms and the gut biome which may explain increased cardiovascular risks in such people (Aho et al., 2016).

## The neurological organization of sleep

Most living organisms, even those without brains, display a regular activity-rest cycle that is synchronized with the solar day. This internal representation of the light-dark cycle enables the organism to adapt itself to its environment, allowing for optimal functioning. Sleep timing is remarkably stable within a given species, highlighting its importance. According to current models, sleep timing arises from the interaction of two inter-related processes: the circadian rhythm often called process C and the homeostatic also known as process S. While both the timing and propensity of REM and NREM sleep is affected by the interaction of these two processes (Borbely, 1982), REM sleep is influenced primarily by the circadian rhythm and NREM sleep is influenced mostly by sleep homeostasis or sleep inertia/pressure. Put another way, the propensity for REM sleep follows clock time while NREM sleep propensity is proportional to the length of prior wakefulness.

The neural substrate for the circadian rhythm is located within paired nuclei of the anterior hypothalamus known as the supra-chiasmatic nuclei (SCN) (Moore, 1992). Autonomic, behavioral, humoral, and temporal regulation mechanisms all contribute to synchronization of the SCN, but the primary stimulus for initiating and maintaining a stable sleep-wake cycle is regular exposure to sunlight. Incident sunlight on the retina in the morning resets the sleep/wake cycle much like setting a mechanical watch after winding it. Light impulses are transmitted *via* retinal ganglion cells by a monosynaptic pathway, the retinohypothalamic tract (RHT) (Dai et al., 1998), to the paired SCN. Projections from the SCN in turn stimulate cells of the paraventricular nucleus (PVN) that project to the upper thoracic portion of the sympathetic nervous system. These neurons innervate cells of the superior cervical ganglion (SCG) that connect to the pineal gland, thereby stimulating secretion of melatonin at sundown in both diurnal and nocturnal species. The SCN has melatonin receptors that provide negative feedback to the SCN-melatonin circuit. Light inhibits melatonin production.

The SCN may be considered the master clock to which other physiologic processes are entrained. Aside from its pacemaker role in sleep-wake activity, this master clock also has enormous influence on locomotor, feeding and endocrine activities. Input from the retinal ganglion cells induces the transcription of specific “clock genes” such as CLOCK (circadian locomotor output cycles protein kaput) and BMAL1 (aryl hydrocarbon receptor nuclear translocator-like protein 1). The approximate 24-h period of the human circadian rhythm is determined by the transcription/translation feedback loop of these “clock genes” and “clock-controlled genes” (CCG). The forward or positive arm of the loop consists of the transcriptional products of CLOCK and BMAL1. A heterodimer of these transcripts activates the transcription of CCG in peripheral tissues whose products eventually return to the cell nucleus to inactivate transcription of CLOCK and BMAL1 in a negative feedback manner (Andreani et al., 2015). The rate and timing of these metabolic processes varies from person to person and determines an individual's chronotype, i.e., being a lark vs. being a night owl. The intrinsic oscillation of this feedback loop is ~24 h. Research over the last few decades has indicated that the SCN may be considered the master clock of a network of biological clocks which reside in almost all peripheral tissues (Takahashi et al., 2008). Further, lesions of the SCN result in loss of its self-sustained oscillations disrupting downstream circadian phenomena (Lehman et al., 1987; Hu et al., 2007).

Clock genes in turn control the transcription of hundreds of CCGs that affect the timing of multiple tissue-specific functions (Korencic et al., 2014). Various genes have circadian rhythms in multiple tissues but often differ in their respective phases. Omic techniques have revealed that widespread oscillation of tissue specific gene transcription occurs in metabolic processes such as carbohydrate metabolism, lipid synthesis, oxidative phosphorylation, and adipocyte differentiation (Zvonic et al., 2006; Bray et al., 2007; Zhang et al., 2014). For example, endocrine and liver function are known to oscillate with a 24-h cycle in a manner that is influenced heavily by nutrient intake The phase relationships among these genes and tissues are not well-understood. Despite the importance of genetic factors in determining the rhythmicity of cellular metabolic activity, behaviors such as feeding can modify said activity (Stokkan et al., 2001; Potter et al., 2016). The metabolic products of feeding/fasting reciprocally interact with the circadian system *via* CCG and the clock genes themselves serving to maintain or disrupt circadian synchrony. Circadian clocks have a strong influence on carbohydrate and lipid metabolism, blood pressure regulation, hormonal secretion, and immune function (Labyak et al., 2002; Scheer et al., 2009; Castanon-Cervantes et al., 2010).

In addition to effects on cellular metabolism, behavioral phenomena change according to circadian oscillations. For example, mental acuity varies with the time of day with two “dips” in mental alertness occurring roughly 12 h apart, from about 1–3 a.m. and again at 1–3 p.m. The latter phenomenon may explain the complaint of sleepiness ascribed to lunch in persons who are probably sleep deprived to some degree. Memory and learning are clearly affected by disruption of circadian rhythmicity (Krishnan and Lyons, 2015). Disruption of SCN rhythmicity in Siberian hamsters is associated with severe deficits in memory while ablation of this structure has little effect on memory (Ruby, 2021).

The biology of process S is less well-understood than that of process C. As mentioned earlier, process S may be viewed as sleep inertia, with which most people are familiar. The longer one is awake, the greater is physiological sleepiness. The best described physiological signature of the homeostatic process is slow wave activity (SWA) seen with standard polysomnography (PSG). Extended wakefulness results in an increase of SWA power with acute recovery sleep. Tumor necrosis factor (TNF) and adenosine may be endophenotypes of sleepiness induced by extended wakefulness. PET (Positron Emission Tomography) scans indicate that adenosine receptors and endogenous adenosine within the brain both increase with extended wakefulness (Elmenhorst et al., 2007). Recovery sleep reverses this phenomenon. Caffeine is a potent inhibitor of adenosine.

While it is helpful conceptually to view process C and process S as distinct, they are two sides of the sleep-wake coin. The interplay of these two phenomena accounts for the timing, length and quality of sleep. Disruption of either the circadian or homeostatic process is generally accompanied by complaints of insomnia and/or excessive daytime sleepiness. Together, the circadian rhythm and homeostatic process have far-reaching effects on all facets of human behavior, human health, and human disease.

## Sleep-related cerebral metabolism

Behaviorally, sleep is characterized by physical inactivity. With respect to the brain, sleep is a highly metabolically active undertaking. The brain undergoes significant changes during sleep, including synaptic reorganization and disposal of waste products that accumulate during wakefulness, allowing it to consolidate new information (Eugene and Masiak, 2015). It is likely that unneeded or redundant synapses are “pruned” during sleep. The synaptic homeostasis hypothesis suggests that salient synapses formed during “learning” are strengthened during sleep while “unimportant” ones probably involute during sleep to conserve energy and maximize the limited space available for the brain (Tononi and Cirella, 2014).

This working hypothesis is given support by focal enhancement of homeostatic sleep related SWA which follows learning of a specific motor task (Huber et al., 2004). Focal SWA power in subjects who had learned a novel visuospatial motor task was significantly increased in the right parietal lobe and was associated with improvement in task performance only if the subject had normal sleep following the learning of the task. Enhanced task performance did not occur in subjects retested following a period of wakefulness. Conversely, this same group demonstrated that immobilization of an extremity resulted in focal reduction in SWA power which correlated with marked reduction in movement efficiency as measured by the variability of the hand-path area normalized to path length (Huber et al., 2006). These findings suggest that sleep “needs” vary locally based on energy demands of synaptic formation/reorganization incurred during learning. Moreover, SWA may represent the electrophysiologic correlate of synaptic plasticity at least under some circumstances. SWA probably reflects synaptic potentiation that is related to cellular requirement of sleep that arises from the metabolic demands of learning (Wilhelm et al., 2011). Energy requirements of synaptic maintenance call for preservation of those connections that are adaptive for optimum functioning.

Recent research in rodents has revealed a system of lymphatics of the brain which likely has an analogous function to the systemic lymphatics of the body. It is posited that this so-called glymphatic system functions to remove toxins from the central nervous system which accumulate during wakefulness (Xie et al., 2013). Neuronal activity results in the production of such putative neurotoxins such as beta amyloid, alpha synuclein and tau. The location of glymphatic channels within the interstitium (ISF) of neuronal tissue allows such substances to be removed *via* convective exchange to the cerebrospinal fluid (CSF) with which the glymphatic system communicates. In rodents, these molecules are removed by transit through aquaporin 4 (AQP4) channels. Compared to the waking state, the functional volume of this glymphatic system increases by roughly 60% during sleep consistent with the notion that it is vital to inhibiting the accumulation of toxic materials within the CNS. This sleep-related volume enhancement results in a 95% increase in clearance (Xie et al., 2013). It is likely that the glymphatic system is involved in removal of adenosine which accumulates during consciousness. While direct evidence for a glymphatic system in humans is lacking, perivascular glial cells in human brains have a similar distribution of AQP 4 as those of rodents. Additionally, AQP 4 channels are reduced in patients with Alzheimer disease but are intact in elderly controls (Zeppenfeld et al., 2016).

The foregoing indicates the CNS is far from metabolically quiescent during sleep. Research of sleep-related brain metabolism has demonstrated increased localized rate of glucose metabolism during both NREM and REM sleep compared to wakefulness. In NREM sleep regions such as the mesial temporal lobes, anterior cingulate gyrus, dorsoparietal association cortex, hypothalamus, pontine tegmentum, and basal forebrain have elevated metabolic rates compared to the waking state (Nofzinger et al., 2002). The mesial temporal lobe contains structures vital to learning and modulation of emotions. Further, accumulation of the aforementioned neurotoxins within the nucleus basalis of Meynert (basal forebrain) is a pathological hallmark of Alzheimer's disease (Whitehouse et al., 1982). If sleep is critical for the removal of such substances from the brain, then chronic sleep disruption/deprivation may represent a significant risk factor for dementia.

The pontine tegmentum, limbic and paralimbic areas demonstrate elevated metabolism during REM sleep according to PET data (Braun et al., 1998). Such increased activity noted also in the extra-striate visual association areas may explain the visual imagery of dreaming during REM sleep. The medial prefrontal cortex (MPFC), retrosplenial cingulate gyrus, parahippocampal gyrus, key components of the default mode network (DMN), likewise displays elevated metabolic activity during REM sleep whereas the salience network is relatively quiescent (Ultermarkt et al., 2020). Slow wave power in sleep is thought to reflect the process of synaptic homeostasis which has been correlated with Process S (Tononi and Cirelli, 2006). Enhanced synaptic strength, occurring during consciousness, is influenced by factors such as active learning and highly salient events and results from the process of long-term potentiation which is thought to underpin neuroplasticity and learning. Process S, according to the synaptic homeostasis model, produces corresponding down regulation of synaptic strength which is supported by empiric reduction of molecular products of LTP which occurs during slow wave sleep (Cirelli, 2001).

## Insomnia

The insomnias represent one of the most common presenting complaints to the primary care physician. Some epidemiological studies indicate that anywhere from 20 to 50% of adults suffer from insomnia (Morin et al., 2011; Bhaskar et al., 2016). According to the latest International Classification of Sleep Disorders, 3rd Ed. insomnia is defined as “a persistent difficulty with sleep onset, maintenance, consolidation, or quality that occurs despite adequate opportunity and circumstances for sleep, and results in some form of impairment of daytime functioning.” The practical effect of insomnia is often insufficient sleep. Multiple clinical studies have confirmed that insufficient sleep impairs all aspects of cerebral functioning including attention, memory, judgement, executive skills, insight and emotional modulation. Sleep deprivation may be viewed as extended wakefulness. A study in 2003 (Van Dongen et al., 2003) demonstrated that individuals allowed only 6 h sleep per night for 2 weeks (chronic extended wakefulness) experienced significant deterioration in all cognitive domains compared to persons with normal sleep time. These subjects were frequently as impaired in some tasks as those with 48 h of extended wakefulness (acute extended wakefulness). As important, the subjects allowed 6 h sleep per night have very little insight into their impairment compared to subjects with acute extended wakefulness. Thus, what may be considered relatively modest sleep restriction has seriously deleterious effects on all aspects of neurocognitive functioning in a dose-dependent manner. The clinical significance of this cannot be overstated, as chronic sleep deprivation is generally considered a benign condition. The practical consequences of insomnia include reduced work productivity, increased incidence of disorders of mood, increased motor vehicle accidents as well as increased cardiometabolic mortality (Vgontzas et al., 2009, 2010; Bertisch et al., 2018).

Historically, insomnia has been viewed from a behavioral perspective, which posits that it occurs in susceptible individuals who experience a precipitating event. Certain psychological traits render some individuals prone to insomnia, which is set into motion by an adverse event such as a death, divorce or job loss. The symptoms of insomnia are then thought to be perpetuated by trait-driven maladaptive coping strategies such as alcohol consumption or staying in bed while awake which may produce an inappropriate conditioned arousal and eventually chronic insomnia (Buysse and Perlis, 1996; Perlis et al., 1997). Multiple endophenotypes associated with insomniacs are all consistently elevated, i.e., dysregulated physiological arousal of the CNS. Abundant clinical evidence has associated insomnia with specific psychological phenotypes, increased activation of the hypothalamic pituitary adrenal system (HPA) and other physiological evidence of elevated arousal. Products of the HPA system e.g., cortisol and epinephrine are associated with stress. Considerable evidence indicates that chronic stress resulting in autonomic imbalance, rather than hypercholesterolemia, is the primary cause of cardiac mortality (Baroldi and Silver, 2004).

Multiple physiological endophenotypes indicate that patients with insomnia have elevated central nervous system arousal. CNS hyperarousal in insomnia manifests as somatic, cognitive and neurocognitive phenomena, but all such arousal must have neurophysiological underpinnings. This model posits that hyperarousal may result from excessive activation of the reticular activating system *via* orexinergic pathways coupled with hypo-functioning of the ventrolateral preoptic nucleus (VLPO), which induces sleep *via* GABAergic and galinergic pathways. Somatic arousal manifests as elevated heart rate, cortisol levels, body temperature, EMG activity of the frontalis and mentalis muscles, skin resistance and whole-body metabolic rate (Bonnet and Arand, 1995, 2010; Vgontzas et al., 2001; Rodenbeck et al., 2002). Further, despite often having shortened total sleep times, insomnia patients often manifest prolonged sleep latencies during the day (Stepanski et al., 1988). Cognitive hyperarousal is reflected in recurrent, intrusive, negative cognitions that are viewed by the patient as being involuntary. When such cognitive patterns are induced experimentally, they result in prolonged sleep latencies in subjects who are partially sleep deprived (De Valck et al., 2004).

EEG activity may be considered an endophenotype of neurocognitive arousal, manifesting as altered power spectra compared to normal sleepers. For example, persons with primary insomnia (PI) do not demonstrate the usual reduction of high frequencies at sleep onset and during sleep maintenance (De Gennaro et al., 2001). They also exhibit increased beta power during waking (Staner et al., 2003), increased beta and gamma during NREM sleep (Lamarche and Ogilvie, 1997), and elevated beta during REM sleep (Perlis et al., 2001). Elevation of these frequencies is seen in states of hyperarousal involved in focused attention and memory (Basar-Ergolu et al., 1996; Jefferys et al., 1996; Makeig and Jung, 1996). Insomnia patients also exhibit increased alpha power in sleep despite having lower absolute alpha power during wakefulness (Merica et al., 1998; Staner et al., 2003). Additionally, patients with PI have significant reduction of delta power during all phases of sleep based on QEEG (Lamarche and Ogilvie, 1997). Oh et al. have recently described increased generalized beta activity in insomnia patients compared to controls (Oh et al., 2020). Heart Rate Variability (HRV) analysis also indicated increased sympathetic tone based on differences in the low frequency (LF) band. Positive correlations were also noted between LF and Pittsburgh Sleep Quality Index (PSQI), Insomnia Severity Index (ISI) and the Beck Depression Inventory. In contrast to prior studies, these findings refer to patients during the day as opposed to the evening or near bedtime (Oh et al., 2020).

Functional neuroimaging has further underscored CNS hyperarousal in insomnia. Compared to normal sleepers, patients with PI have smaller differences in metabolic rates between wake and NREM in areas such as the DMN (Nofzinger, 2004; Kay et al., 2016). Further, PET scans have also shown a significant increase in metabolism in the whole brain (Nofzinger, 2004), but relative reduction in the DMN in insomniacs compared to controls (Kay et al., 2016). This latter finding is compatible with hyperarousal, inasmuch as the DMN is thought to be more active during mental quiescence compared to directed task management.

The foregoing data indicating generalized hyperarousal in insomniacs suggest that it results from CNS dysregulation, which may impinge on daytime functioning apart from its effects on sleep quality *per se*. A dysregulated brain may thus create clinical phenotypes in many areas of normal functioning apart from those that may be attributed solely to neuropsychological disorders.

## Treatment of insomnia

Hypnotic medications remain the most common recommended therapy for insomnia. Numerous medications are prescribed for symptoms of insomnia, many of which are taken for years despite lack of evidence of clear benefit under such circumstances. Millions of prescriptions for hypnotics are written annually, often for older adults.

The 2017 American Academy of Sleep Medicine (AASM) guidelines for the pharmacologic treatment of insomnia using the “GRADE” process classifies the current evidence for available hypnotic medications as WEAK. “These recommendations are based on a systematic review of available randomized controlled trials of individual drugs, taking into consideration the quality of evidence, risk-benefit ratio, and patient preferences. A STRONG recommendation is one that the clinician should, under most circumstances, follow, whereas a WEAK recommendation indicates a lower degree of certainty in the outcome and appropriateness of the patient-care strategy for all patients. Downgrading the quality of evidence in this process reflects such factors as funding sources for most clinical trials and associated publication bias and the small number of eligible trials” (Sateia et al., 2017).

It should be noted that the FDA has issued multiple Drug Safety Communications regarding pharmacotherapy for insomnia. These warnings describe potential morbidity from next-day impairment associated with use of zolpidem (2013) and eszopiclone (2014), resulting in the lowering of recommended dosing particularly for women. The zolpidem warning came after more than two decades of market experience. They have also described risk of serious injury or death with the combination of benzodiazepines and opiates (2016). Interestingly, the FDA warned against withholding opioids from addicts also taking other CNS depressants.

FDA warnings have been empirically validated by epidemiologic studies with demonstrate elevated all-cause mortality correlated with hypnotic drug usage. This is probably attributable to respiratory depression, especially when used in combination with alcohol or opiates. Multiple illnesses such as cancer, accidents, serious infection, and disorders of mood have elevated hazard ratios (HRs) of death associated with hypnotic usage (Kripke, 2018). Sun has reported clinically relevant increase in suicide among patients with chronic zolpidem usage, the odds ratios (ORs) ranging from (1.90–2.98) being dependent on total dose exposure (Sun et al., 2016). Using a model based on redefining unintentional suicide due to drug intoxication, Rockett reported 68,298 such deaths in 2013, a number exceeding that due to motor vehicle accidents (Rockett and Caine, 2015). Other studies have shown that mortality HRs are proportional to total doses of hypnotic medication per year. For example, according to the Geisinger Health System report adjusted mortality ratios correlated directly with total dose exposure: 3.60 for 4–18 doses per annum, 4.43 for 19–132 doses and finally, 5.32 for >132 doses (Hartz and Ross, 2012). Statistical extrapolation of the Geisinger data to the entire population of hypnotic users in the United States resulted in an estimated annual mortality of ~300,000–500,000, more than is attributed to tobacco usage.

In its most recent clinical guidelines for behavioral and psychological treatment of chronic insomnia in patients with or without psychiatric or medical co-morbidities, the AASM (American Academy of Sleep Medicine) gives a STRONG recommendation for cognitive-behavior therapy (CBT). This recommendation was based on a large body of moderate quality evidence from 49 studies (Edinger et al., 2021). The premise of CBT is that many psychological disorders are driven by maladaptive thought processes that lead to emotional distress, physiological over-arousal and learned dysfunctional behaviors. Given the previous discussion of the potential mechanisms of insomnia, CBT clearly has plausible therapeutic potential. Examples of dysregulated cognition in patients with insomnia may include unrealistic expectations regarding oneself, family and friends or even world events that are specific to a given patient. CBT employs psychotherapeutic strategies to neutralize the dysregulated cognition that is thought to lead to emotional distress, elevated arousal and maladaptive behaviors. It is a mentally intensive process that requires active patient participation in concert with an experienced practitioner who will appropriately challenge the validity of the patient's dysfunctional beliefs.

Okajima et al. (2011) has examined the clinical utility of CBT-I (CBT for insomnia) in a meta-analysis that included 14 clinical trials published from 1990 to 2009. Effect sizes of CBT-I on multiple parameters of sleep such as sleep latency, sleep efficiency and total sleep time in patients with insomnia were medium to large on many of these parameters until the effect of publication bias was considered. With this factor removed significant effect sizes of CBT-I remained in only a few of the studied parameters.

## Behavioral conditioning in neurological disease

The use of behavioral modification as therapy for neurological disorders has been described for more than 200 years. Lysons described a patient with focal epilepsy that began with altered sensations of one foot which traveled proximally, and the patient would lose consciousness when these paresethesias reached his knee. He learned that the tight application of a garter at the knee progressively abated the fits until ultimately he required no treatment (Lysons, 1772). Likewise, Gowers noted in a patient with Jacksonian epilepsy whose seizures began in the hand that the fits could be abolished with the application of a tourniquet located proximal to the elbow (Gowers, 1881).

Conditioning stimulation has been used to elicit specific physiological responses, including EEG activity, proving that behavioral conditioning has direct effects on neurophysiology i.e., self-regulation. Voluntary (non-reflexive) miosis, for example, has been coupled with a specific sound, thence to the command to “constrict” (Hudgins, 1933). Eventually, subjects are able to produce pupillary constrictions just by thinking of the command. Other researchers have successfully conditioned alpha blocking to non-visual stimuli and as with miosis, to the mere thought of the stimulus (Travis and Egan, 1938; Jasper and Shagass, 1941a,b). The physiological underpinnings of these phenomena are the same as those used by Pavlov to elicit salivation in dogs conditioned to the sound of a bell. Such techniques may be used to activate neural pathways—both inhibitory and excitatory—and thereby disrupt either a normal physiological response or a pathological one.

An elegant case of such conditioning, in a 41-year-old professional singer who experienced a 26-year history of rigidly stereotypical uncinate epilepsy, was presented by Efron (1956, 1957). The spells were heralded by an extended and intense sensation of depersonalization followed closely by the compulsive thought of an expected odor that never happened. In turn, she then experienced a noxious olfactory hallucination, an auditory hallucination, versive head movement culminating in a convulsive epileptic seizure. Beginning about the age of 15 years she experienced ~7–15 spells monthly with no more than a fortnight of reprieve. The condition had been intractable to both diphenylhydantoin and phenobarbital.

Efron successfully aborted the seizures using an olfactory stimulus of jasmine, which had to be applied prior to the appearance of the forced thinking. The effect of this olfactory stimulus was then successfully transferred to the viewing of a silver bracelet demonstrating a second order conditioned response. Finally, the patient could terminate the seizures with a mental image of the bracelet, which was then always accompanied by the phantom odor of jasmine.

Following this conditioning therapy, she was exposed to I.V. metrazol (Fabing, 1942) to document the correlation of her semiology with progressive changes in her EEG. Surprisingly, she had become resistant to high doses of this pro-convulsant. During the subsequent 6 weeks she arrested the progression of her seizures by visualizing the bracelet. The patient had no spells of depersonalization for 14 months, rather only occasional experiences of the phantom aroma of jasmine. Having declared herself cured, she resumed her career of singing.

The foregoing clearly demonstrates that the adult brain is plastic and capable of reorganizing itself with behavioral conditioning to optimize its self-regulatory capacity. How it accomplishes this is poorly understood at best, despite abundant clinical and scientific evidence that it can do so. Harnessing the brain's plasticity through directing its own endogenous modulation now holds promise to provide a non-invasive, safe, and effective therapy for many conditions for which modern medicine has little to offer.

## Insomnia as a manifestation of CNS dysregulation

Functionally, the CNS may be viewed as a highly regulated complex, interconnected, hierarchical system of networks. While an engineering explanation of brain functioning has some utility, it falls far short of explaining the totality of phenomena described in both experimental and clinical “neuropsychology.” With the acknowledgment that we understand little about the causal mechanisms of human psychology i.e., exactly how the brain does what it does, we may proceed with the following. A useful model of brain self-regulation invokes several primary characteristics. At the most fundamental level, the CNS must have a stable level of arousal, which underpins all aspects of brain functioning that require appropriate vigilance and upon which adaptive behavior depends. Interconnected to the level of arousal are autonomic and affective tone. These three factors largely determine the functional state of the CNS acutely and chronically. Thus, it is not surprising that one's ability to sleep well is dependent on these issues.

As alluded to previously, impaired cerebral self-regulation leads to chronic illness with attendant morbidity and mortality. For example, chronic dysregulation of autonomic tone is very likely the major contributor to heart attack (Baroldi and Silver, 2004). Furthermore, the timing of heart attacks demonstrates a clear circadian effect (Buurma et al., 2019; Crnko et al., 2019). Also, the symptoms of certain chronic disorders such as epilepsy and asthma are frequently related to sleep (Cukic et al., 2011; St. Louis, 2011). Inability to modulate affect underpins psychological disorders such as anxiety and depression and developmental disorders like autism. As we have shown, somatic and neurocognitive hyperarousal is the basis for primary insomnia. Neurofeedback addresses these core difficulties with self-regulation training that helps to promote good sleep.

## Neurofeedback and sleep

Exploiting and engaging the subconscious self-regulatory mechanisms explains much of what has been documented with neurofeedback, especially with infra-low frequency (ILF) neurofeedback. The first clues of this phenomenon in “modern” times occurred in 1970 when Sterman described (Sterman et al., 1970) the potentiation of sleep spindles (frequency ~12–14 Hz) in cats by way of classical conditioning to produce a similar waking EEG pattern, located over the sensorimotor cortex, which he labeled the sensorimotor rhythm (SMR). Roth had associated this specific EEG pattern with the inhibition of conditioned motor responses (Roth et al., 1967). Sterman's cats also demonstrated longer periods of consolidated sleep characterized by less frequent motor activity typically fragmenting the quiet sleep of cats. Thus, this operant EEG conditioning was thought to represent a meaningful change in neurophysiology during waking and sleep.

Sleep spindles, an endophenotype of thalamocortical activity, result in reduced activity of gamma motor neurons in the cat (Hongo et al., 1963). Furthermore, spinal cord injured patients exhibit increased SMR that results from decreased afferent neuronal activity to the thalami from the affected extremities. Some patients with epilepsy have severe reduction of both SMR and sleep spindles, which increases following SMR conditioning (Sterman, 1977). Spindle-burst density during NREM sleep correlates directly with auditory threshold (Bonnet and Moore, 1982). Additionally, subpopulations of sleep spindles may be targeted by differential sound oscillations, 11–13.5 Hz frontally, 13.5–16 Hz posteriorly, to alter the distribution of fast and slow spindles arising from the parietal regions (Anthony and Paller, 2016). These phenomena strongly suggest that spindle bursts, especially of N2 sleep, constitute a filter or gating mechanism of sensory input and motor output of the CNS. Depending on afferent input, spindle activity may be the physiological representation of blockade of such input to the thalamus, which structure is necessary for corresponding motor output such as walking on the one hand or convulsive epilepsy on the other.

Most clinical studies using neurofeedback in insomnia involve only patients with primary or psychophysiological insomnia. Such individuals have no medical or physiological condition known to cause insomnia. That said, these subjects do have biochemical, physiologic and neurophysiologic evidence of elevated arousal. A recent study which will be discussed later by Carlson et al. has described significant improvement in insomnia in patients with PTSD (Carlson and Ross, 2021).

Clinically relevant improvement in insomnia was first noted by Budzynski, who reported that reduction in the sleep latencies of 6 of 11 insomniacs after a combination of alpha training, theta training and EMG training of the frontalis muscle. His exact protocol was not described, making it impossible to know which of the therapies was beneficial (Budzynski, 1973).

Improvement in self-reported sleep indices including time to settle down to sleep, pre-sleep intrusive thoughts, estimated sleep latency, number of awakenings, and total sleep time occurred in a 42-year-old healthy woman with no prior history of psychiatric disorder following 11 sessions of theta neurofeedback. She had taken 5 mg of nitrazepam nightly for 5 years prior to the therapy. Her sleep aid was withdrawn over the 2 weeks following the NF (Bell, 1979).

By the end of her therapy her theta density increased from 9% at baseline to 17%. Aside from her subjective improvement in sleep, she also reported improvement in her sense of wellbeing based on the short version of the General Health Questionnaire. At 3 months follow-up she had maintained her improvement.

Similarly, Hauri noted treatment-specific improvement in patients with psychophysiologic insomnia depending on whether the subject exhibited high somatic or psychological tension. The former responded better to theta feedback whereas the latter responded only to SMR feedback (Hauri, 1981).

In a small, randomized trial using standard protocols for each type of neurofeedback, something not done initially, Hauri replicated his earlier findings. The patients underwent training with either theta or SMR neurofeedback for 26 sessions over 13 weeks. It is noted that during SMR training, theta was inhibited, the converse being the case with theta training. Surprisingly, all patients improved in subjective sleep experience irrespective of the type of training, but only those who received “appropriate” training showed objective improvement on PSG. At 9 months follow-up improvements had been maintained (Hauri, 1982).

Berner et al. (2006) examined the effect of four 10-min neurofeedback sessions targeted to reinforce 11.6–16 Hz activity administered 3 h prior to bed on both sleep spindle generation and a declarative memory test the following day. There was no effect on the density of sleep spindles *per se*, but noted was a slight increase in sigma power during NREM in experimental subjects compared to those who received sham feedback. However, performance on a paired association test did correlate with spindle density with good performers having on average 33% higher spindle activity compared to poor performers.

The same group undertook a follow-up study (Hoedlmoser et al., 2008) that demonstrated increased sleep spindles and shortened sleep latency after 10 24-min sessions of SMR training on consecutive days. The subjects also improved performance on a word pair association task compared to those who received random frequency feedback. Wakefulness training resulted in transference of EEG activity during sleep just as was the case with the cats trained by Sterman.

To examine the presence of neurocognitive hyperarousal in insomnia, Cortoos undertook a comparison of tele-neurofeedback to tele-EMG biofeedback in 17 subjects with primary insomnia specifically to affect EEG patterns associated with cognitive processing, namely high beta frequencies (Cortoos et al., 2010). Importantly, theta frequencies were inhibited as these are associated with reduction of daytime cognitive performance (Klimesch et al., 2007). The NF group trained SMR (12–15 Hz) with theta and high beta inhibition at Cz. Conversely, the biofeedback subjects were instructed to reduce EMG activity at Fpz, considered tantamount to up-regulation of relaxation. Both groups underwent 20 20-min sessions with identical visual displays over 8 weeks. Compared to the biofeedback group, the NF subjects experienced increased total sleep time and overall improvement in sleep log data completed at home. Both groups had shortened sleep latencies compared to baseline sleep studies.

Real-time Z-score SMR training was compared to QEEG-guided training in a study randomizing four patients to each arm (Hammer et al., 2011) which consisted of 15 20-min sessions. Improvement in sleep efficiency as measured by the Pittsburgh Sleep Quality Index (PSQI) was a primary end point. Additional pre- and post-assessments included the Psychiatric Diagnostic Screening Questionnaire, the Minnesota Multiphasic Personality Inventory-2-Revised Form, the Insomnia Severity Index (ISI), the Psychiatric Diagnostic Screening Questionnaire, and the Quality-of-Life Inventory. Subjects were free of over the counter and prescription medications for insomnia. Caffeine was limited to five cups of coffee or other caffeinated beverages per day. Shift workers and patients with mental or medical disorders which could affect sleep were excluded.

Results indicated improvement in all sleep parameters in both groups regardless of mode of NF. Both groups had mean increase in sleep efficiency of 16%. All had normal post-treatment sleep efficiencies with the mean being 93%. Improvement in measured sleep indices was coupled with comparable improvements in overall sense of wellbeing, mood and quality of life. These findings strongly indicate that the more accessible and simpler SMR training was as effective as QEEG-guided NF.

A counterbalanced within-subjects-design with 24 subjects with PI who were randomized to SMR instrumental conditioning (ISC) at C3 or to pseudo-instrumental conditioning (PFT). All were screened with validated tools to exclude psychiatric or medical conditions. Further, subjects were free of medications/drugs and caffeine and nicotine usage was limited. All subjects met AASM criteria for PI. Target frequency in the active arm was 12–15 Hz consisting of 8 3-min blocks of conditioning for a total of 10 sessions. PFT consisted of 5 sessions that were otherwise identical to the experimental group with reinforcement targeting 7–20 Hz exclusive of 12–15 Hz. While subjects were instructed to move a needle as far left as possible, they were given no other information. A minimum reward incidence of 5 per 3-min session was given to maintain appropriate motivation in the PFT group. Both groups underwent standard PSG at baseline and post-treatment (Schabus et al., 2014).

ISC subjects demonstrated increased N3 with fewer overall awakenings on post-treatment PSG compared to PFT subjects. The increased N3 was also associated with a large enhancement of fast spindles in these subjects. Post-treatment PSQI (M = 10.8 pre and M = 7.00 post) indicated improvement of sense of wellbeing. All subjects demonstrated baseline significant overnight forgetting with no overall change in either experimental arm post-treatment. However, linear correlations suggested an association between improvement in memory and individual ISC success. The authors posited that the degree of 12–15 Hz enhancement was correlated with overnight memory consolidation, which they attributed to increased fast spindle density. It is thus plausible that with more training, an unambiguous improvement on memory may become evident like the findings of Hoedlmoser. The data also indicated that PFT had negative consequences on sleep.

A more recent study by Schabus et al. (2017) concluded that neurofeedback had no effect on insomnia patients compared to placebo despite their earlier works that reached other conclusions (Berner et al., 2006; Hoedlmoser et al., 2008; Schabus et al., 2014). The latest work had a similar design but failed to include inhibit training despite ostensibly following their earlier efforts. Lack of control for inhibits could have resulted in rewarding non-SMR phenomena. For example, a dysregulated brain may produce maladaptive bursts in the 12–15 Hz range that will be rewarded if appropriate inhibits are not in place. A similar risk prevails with bursts of EMG at the reference electrode. Moreover, inhibit-based controls do, in and of themselves, enhance the brain's regulatory competence. Absence of appropriate inhibit training could be sufficient to result in the lack of beneficial effects described in this study. Finally, based on the collective experience within the field of neurofeedback, rewarding frequencies other than SMR would be expected to have some effect on brain self-regulation and in no wise could have been accounted for in this study. One may conclude that both arms of the crossover design were active interventions.

In 2021 Carlson and Ross (2021) demonstrated marked improvement in four veterans with confirmed minor traumatic brain injury (mTBI) and typical post-concussion symptoms including insomnia, following infra-low frequency (ILF) neurofeedback (Othmer et al., 2013; Othmer and Othmer, 2016) using the Othmer protocol (2019 Protocol Guide). This pilot study utilized an unconventional approach referred to as infra-low frequency (ILF) NFB, which is also the impetus for this paper. The method employs feedback from the Slow Cortical Potential (SCP) domain, comprising frequencies below 0.1 Hz and frequently as low as 0.1 milli-Hz (Othmer and Othmer, 2020; Cygnet https://www.beemedic.com/bee-medic-technologies-in-mental-health.aspx).

ILF NFB exploits latent neuroplasticity through real-time feedback on the time course of the frequency-dependent, narrow-band filtered Slow Cortical Potential derived from a bipolar montage, e.g., T3-T4. This differential signal becomes actionable as soon as it is recognized, becoming part of the brain's ongoing project of bringing closure between the observed reality and its intentions. This can be understood as a continuous error-correction process, analogous to what happens when a missile is converging on its target. This process alters relationships within the Intrinsic Connectivity Networks, particularly the DMN and the Salience networks. Identification of this “error signal” provides the basis for the cerebrum to adjust its activity level, moderate neuronal excitability, and alter connectivity relationships. At baseline, mean ISI score of the four veterans was 22.75 indicating severe complaints of insomnia. Post treatment scores were 3.25 consistent with no clinically significant insomnia symptoms. Significant and clinically relevant improvements were also seen in all 12 instruments used to assess patients with mTBI.

The foregoing discussion underscores objective physiological perturbations in people with insomnia which may be considered a manifestation of cerebral dysregulation with important effects on daytime functioning as well as general health. It also briefly reviews a 200 plus years of experience of the benefit of behavioral conditioning in neurological disorders such as epilepsy prior to the availability of modern therapies. The foundation for the evaluation and treatment of insomnia is symptom based. The recent introduction of instrumental conditioning has demonstrated clear benefits in the symptoms of insomnia without the serious limitations of currently available pharmacotherapy. Furthermore, Carlson's subjects demonstrated global improvement in cerebral functioning which underscores the utility of ILF neurofeedback in optimizing brain self-regulation.

The model of CNS regulatory hierarchy developed through the empiric results of neurofeedback involves regulation of arousal, affect and autonomic functions which are commonly disturbed in persons with insomnia. ILF neurofeedback has demonstrated effectiveness in this regulatory hierarchy. Disorders of arousal such as insomnia likely impact Process S. Improved autonomic stability may be responsible for anecdotal reports of improvement in conditions such as sleep terrors and sleep apnea. The same could be said for improved sleep organization being associated with improvement in sleep enuresis, sleep paralysis and somnambulism for which there is also anecdotal evidence. A therapy capable of addressing such conditions, even if not universally successful, speaks strongly in favor of this model and such therapy's ability to optimize what may be viewed as core self-regulation or autonomy.

A future area of clinical research could involve circadian rhythm sleep-wake disorders which to date has not yielded to ILF. Ideally, this would involve correlating available endophenotypes such as circadian temperature variation, melatonin secretion, qEEG abnormalities, and previously described metabolic derangements with clinical symptoms in these patients.

ILF NFB has been in use in our neurology practice for nearly 10 years in application to a variety of conditions normally seen in a neurology practice. The following case report, consistent with the foregoing review of the treatment of insomnia with neurofeedback, illustrates what can be accomplished in application to chronic insomnia.

## Case presentation

A 33-year-old female engineer presented with complaints of headaches, anxiety and insomnia. The initial ISI score was 25, consistent with severe clinical insomnia. She also reported at least three moderately severe to severe headaches per week that had negative effects on her daytime functioning. Sleep onset was also inhibited by frequent mental rumination. She had tried multiple medications including prescription and over the counter sleep aids and prescription mood stabilizers but did not like the associated side effects, some of which she judged to worsen her symptoms. She rated her overall quality of life as below average to poor because of her symptoms. Her general medical history was non-contributory. Initial symptom tracker of 12 complaints yielded a composite score of 110 using a Likert scale of 0–10 per category (EEG Expert, www.eegexpert.net).

She began treatment with Cygnet ILF at T4-P4 initial frequency 0.5 mHz, which was optimized to 0.001 mHz over about 10 sessions. Persistent intrusive and negative thoughts prompted addition of T4-Fp2 for about 8 sessions. She noted improvement in headaches and overall sleep within 6 sessions. After 15 sessions she underwent Alpha-Theta training, following which all her symptoms improved, especially anxiety. She has completed 30 ILF sessions and two Alpha-Theta sessions. Her composite symptom tracker score is now 58 (53% of the starting value). She reports that her headaches have completely resolved, and her ISI is 11, indicative of subthreshold insomnia.

## Conclusions

The foregoing striking case report is just one piece of evidence that supports the conviction, emerging out of our collective clinical practice, that recommends NF as a potentially powerful therapy for a large array of neuropsychological disorders such as insomnia for which current therapies seldom provide functional recovery, are expensive, and are frequently associated with significant morbidity. The method exploits our inherent neuromodulatory capabilities, with the activity at the optimal response frequency acting as a pathfinder for the brain to enhance its self-regulatory competence. Moreover, the beneficial effects of NF are typically long-lasting, once sufficient training has been done. Learning will have occurred, which is then reinforced through the activities of living. To date, there is no evidence that this therapy is associated with long-term adverse effects. NF has a large and growing body of clinical data demonstrating its effectiveness. These data have accumulated over the course of nearly 50 years for NF generally, and over the course of 16 years in the case of ILF NF. NF effects on brain functioning are helping to clarify how self-regulatory capacities are organized. Advances in NF have come about through the individual and collective efforts of professionals with a broad range of backgrounds, allowing researchers to approach this therapy without the usual prejudices that hinder more narrowly specialized fields of scientific inquiry.

## Author contributions

The author confirms being the sole contributor of this work and has approved it for publication.

## Conflict of interest

The author declares that the research was conducted in the absence of any commercial or financial relationships that could be construed as a potential conflict of interest.

## Publisher's note

All claims expressed in this article are solely those of the authors and do not necessarily represent those of their affiliated organizations, or those of the publisher, the editors and the reviewers. Any product that may be evaluated in this article, or claim that may be made by its manufacturer, is not guaranteed or endorsed by the publisher.
